# The anti-cancer effect of retinoic acid signaling in CRC occurs via decreased growth of ALDH+ colon cancer stem cells and increased differentiation of stem cells

**DOI:** 10.18632/oncotarget.26157

**Published:** 2018-10-05

**Authors:** Shirin R. Modarai, Anindita Gupta, Lynn M. Opdenaker, Ryan Kowash, Gabriel Masters, Vignesh Viswanathan, Tao Zhang, Jeremy Z. Fields, Bruce M. Boman

**Affiliations:** ^1^ Department of Biological Sciences, University of Delaware, Newark, DE, USA; ^2^ Center for Translational Cancer Research, Helen F. Graham Cancer Center and Research Institute, Christiana Care Health System, Newark, DE, USA; ^3^ Department of Biological Sciences, Dickinson College, Carlisle, PA, USA; ^4^ Biochemistry Department, Hamilton College, Clinton, NY, USA; ^5^ Genetic and Preventive Medicine, Thomas Jefferson University, Philadelphia, PA, USA; ^6^ Research Pediatric Development, Children's Hospital of Philadelphia, Philadelphia, PA, USA; ^7^ Cancer Research, CATX Inc., Gladwyne, PA, USA

**Keywords:** ALDH, retinoic acid, colorectal cancer, stem cell, neuroendocrine cell

## Abstract

**Background:**

Tumorigenesis is driven by stem cell (SC) overpopulation. Because ALDH is both a marker for SCs in many tissues and a key enzyme in retinoid acid (RA) signaling, we studied RA signaling in normal and malignant colonic SCs.

**Hypothesis:**

RA signaling regulates growth and differentiation of ALDH+ colonic SCs; dysregulation of RA signaling contributes to SC overpopulation and colorectal cancer (CRC) development.

**Methods:**

We analyzed normal and malignant colonic tissues and CRC cell lines to see if retinoid receptors (RXR & RAR) are exclusively expressed in ALDH+ SCs, and if RA signaling changes during CRC development. We determined whether RA signaling regulates cancer SC (CSC) proliferation, differentiation, sphere formation, and population size.

**Results:**

RXR & RAR were expressed in ALDH+ colonic SCs, but not in MCM2+ proliferative cells. Western blotting/immunostaining of CRCs revealed that RA signaling components become overexpressed in parallel with ALDH overexpression, which coincides with the known overpopulation of ALDH+ SCs that occurs during, and drives, CRC development. Treatment of SCs with all-trans retinoic acid (ATRA) decreased proliferation, sphere formation and ALDH+ SC population size, and induced differentiation along the neuroendocrine cell (NEC) lineage.

**Conclusions:**

Retinoid signaling, by regulating ALDH+ colonic CSCs, decreases SC proliferation, sphere formation, and population size, and increases SC differentiation to NECs. Dysregulation of RA signaling in colonic SCs likely contributes to overpopulation of ALDH+ SCs and CRC growth.

**Implications:**

That retinoid receptors RXR and RAR are selectively expressed in ALDH+ SCs indicates RA signaling mainly occurs via ALDH+ SCs, which provides a mechanism to selectively target CSCs.

## INTRODUCTION

Stem cell (SC) overpopulation drives colorectal cancer (CRC) development and growth. Because aberrant regulation of SC dynamics likely contributes to SC overpopulation, understanding how colon SCs are regulated is important [1]. We discovered that aldehyde dehydrogenase (ALDH) is a marker for normal and malignant human colon SCs and tracks SC overpopulation during colon tumorigenesis [2]. Because ALDH is a key enzyme in the retinoic acid (RA) signaling pathway, and because aberrant RA signaling alters cell proliferation and differentiation that contributes to CRC progression [3–5], we studied RA signaling in normal and malignant colon SCs. Our long-term goal was to determine how RA signaling regulates SC dynamics and how dysregulation of RA signaling leads to CRC initiation and progression.

RA signaling is mediated by two nuclear retinoic receptor subtypes: the retinoic acid receptor (RAR) and the retinoic X receptor (RXR) [6]. As both RXR and RAR are involved in dimerization involving different RAR and RXR receptor subtypes, they are the mediators of transcriptional responses to stimuli from multiple kinds of signaling. Both RXR and RAR receptors interact with multiple co-activator and co-repressor proteins to promote increased cell stemness or cell differentiation [6–9].

Two of the most common, naturally occurring retinoic acid ligands that mediate cellular RA signaling are all-trans retinoic acid (ATRA) and 9-cis RA [6]. These ligands are known to have different affinity for RA receptors. Specifically, RAR subtypes have a higher affinity to and become activated by ATRA than other RA ligands [10, 11]. 9-cis RA binds to both RXR and RAR receptors but with higher affinity to RXR [10]. ALDH1, a SC marker, is a key enzyme of the RA signaling pathway that converts retinaldehyde to ATRA, a derivative of retinoic acid. Identifying the role of the RA signaling pathway in colonic SCs and alterations in RA signaling in CSCs might suggest novel targets for cancer therapeutics, particularly since RA agents, such as ATRA, are clinically available.

In the current study, we analyzed colon cancer cell lines and CRC patient tumor samples to determine if changes in key components of the RA pathway, particularly in RXR and RAR, occur during CRC development and progression. Other experiments were designed to determine: 1) if RA receptors (RXR & RAR) are selectively expressed in colonic SCs; 2) if RA signaling regulates stemness and maturation of colonic SC, and 3) if alterations in RA signaling occur during, and contribute to CRC development. Because NECs mainly reside in the crypt SC niche and can regulate the colonic SCs characteristics [12], we also determined if there was a link between RA receptor signaling and SC maturation toward a neuroendocrine cell (NEC) phenotype. Our immediate objective was to determine whether RA signaling regulates colonic SCs and if so, how dysregulation of RA signaling might lead to CRC progression.

## RESULTS

### Expression of RAR, RXR and ALDH in normal colonic epithelium (NCE) and CRC tissues

**Figure 1:**
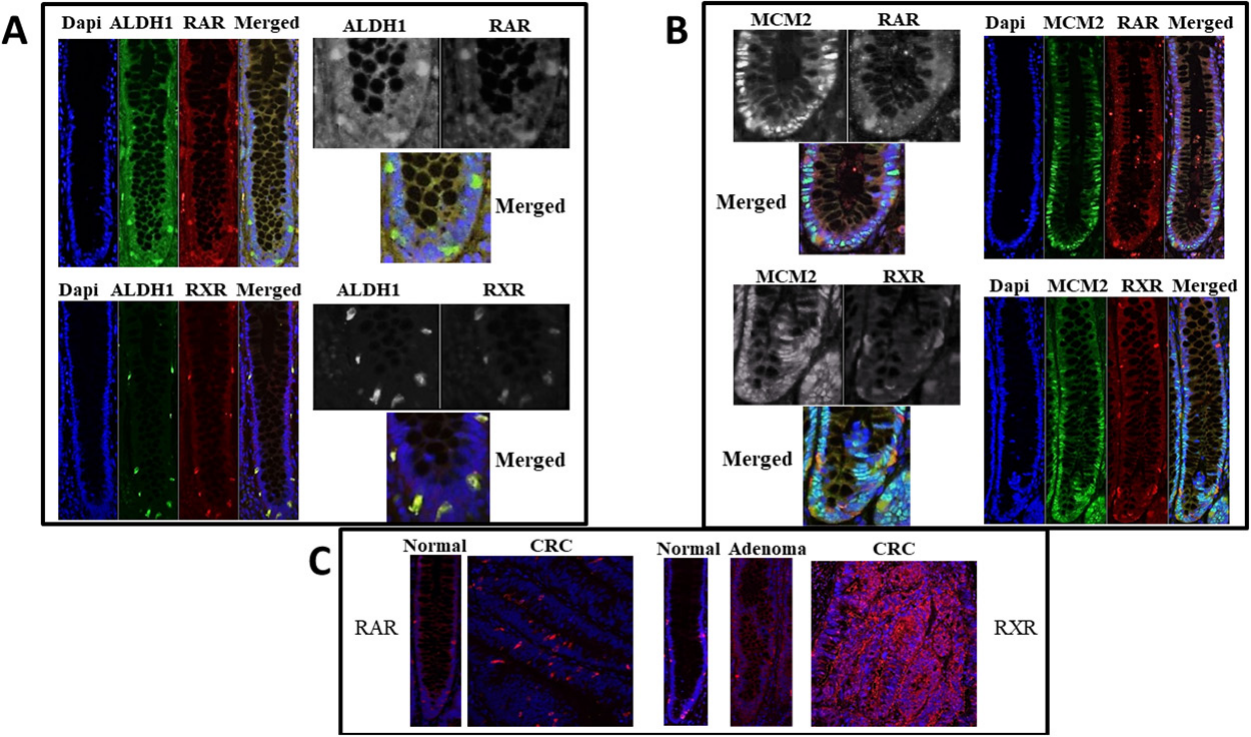
RAR and RXR-alpha retinoid receptors co-stain with ALDH1 but not with MCM2 Normal colonic crypt specimens from paraffin embedded tissue sections were stained which showed co-localization of retinoid receptors with the colonic SC marker ALDH1 (Images in Panel **A**), but not with the proliferating cell marker MCM2 (Images in Panel **B**). To appreciate cell localization of the signal, all single channels are also shown in black and white and merged channels are shown in color at higher magnification. Images in Panel **(C)** show staining (red) for retinoid receptors in colon tumors compared to normal colonic epithelium.

Expression of retinoid receptors RAR and RXR was analyzed in paraffin-embedded tissue sections of normal colonic tissue. We found that the cells staining positively for RAR and RXR were selectively located in the bottom 1/3 of the colonic crypts, particularly in the SC niche. The majority of the ALDH1+ (85%), RAR+ (87%), and RXR+ cells (63%) were located in the SC niche (crypt levels 1-20; [2]). Co-staining for the SC marker ALDH1 and retinoid receptors showed that the majority (>90%) of ALDH+ cells in the SC niche co-express ALDH1 with RAR or RXR (Figure 1A). In comparison, co-staining for ALDH and the cell proliferation marker MCM2 did not show any overlap and the staining patterns identified distinct cell types (Figure 1B).

To determine if expression of retinoid receptors (RXR and RAR) becomes altered during CRC development, immunostaining was also done on normal colonic epithelium (NCE) and CRC tissue samples. CRC tissue samples showed increased ALDH1, RAR, and RXR protein expression as compared to matching normal tissues (Figure 1C). We previously observed a similar alteration for ALDH1 overexpression in CRC progression [2].

**Figure 2:**
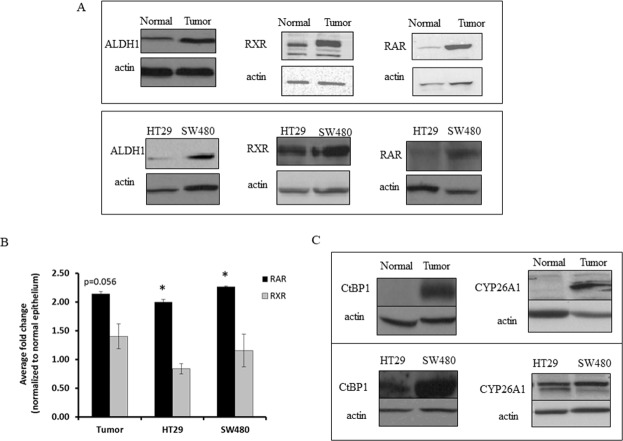
CRC tissue and CRC cell lines express higher levels of ALDH1 and retinoid receptors than normal colonic tissue sections **(A)** Cell lysates were collected and equal protein concentrations were run on an SDS-PAGE gel and in western blot analyses. The representative images show increased expression of retinoid signaling pathway proteins and ALDH1 in tumor tissues and HT29 and SW480 CRC cell lines, as compared to matched normal tissues. **(B)** Densitometry on western blot analysis represents the average fold change values with increased expression of RAR in tumor tissues and CRC cell lines, as normalized to the normal colonic epithelium. The densitometry on western blot analysis represents the average fold change values of RXR in the tumor tissues and CRC cancer cell lines, as normalized to the normal colonic epithelium. This experiment was done in triplicate and error bars represent ± SEM, ^*^ p < 0.05. **(C)** Representative western blot images show differential expression of retinoid signaling pathway proteins CtBP1 and CYP26A1, between HT29 and SW480 cells, and these proteins are almost absent in normal colonic epithelial tissues as compared to the matched tumor tissues.

Given that retinoid receptors and ALDH are key components involved in RA signaling, we also assessed other components of the retinoid signaling pathway using western blot analysis of matched NCE and CRC tissue samples. In parallel with increased ALDH1, RXR and RAR overexpression was seen in CRC as compared to NCE (Figures 1C & 2A, 2B). CRC samples also showed increased expression of CYP26A1 and CtBP1, both of which are downstream of ALDH in the retinoic acid pathway (Figure 2C). Thus we observed increased expression of retinoid receptors, as well as of other key components of the RA pathway in CRC tissues.

### Expression of RAR, RXR, ALDH and retinoid signaling components in CRC cell lines

To understand the relationship between ALDH1 and retinoid receptors, an *in vitro* tissue culture system was employed. Based on previous screening of colon cancer cell lines for ALDH1 expression and ALDH activity, we chose HT29 and SW480 cells [13]. These two cell lines were screened for protein expression of RAR, RXR, ALDH1, CYP26A1 and CtBP1 (Figure 2A-2C). Western blot analysis showed that both cell lines express relatively high levels of all components of the retinoid signaling pathway (Figure 2A-2C). Immunocytochemical staining of HT29 and SW480 cells showed increased RAR and RXR-alpha expression in both lines but SW480 cells had relatively more retinoid receptor positive cells (Supplementary Figure 1).

**Figure 3:**
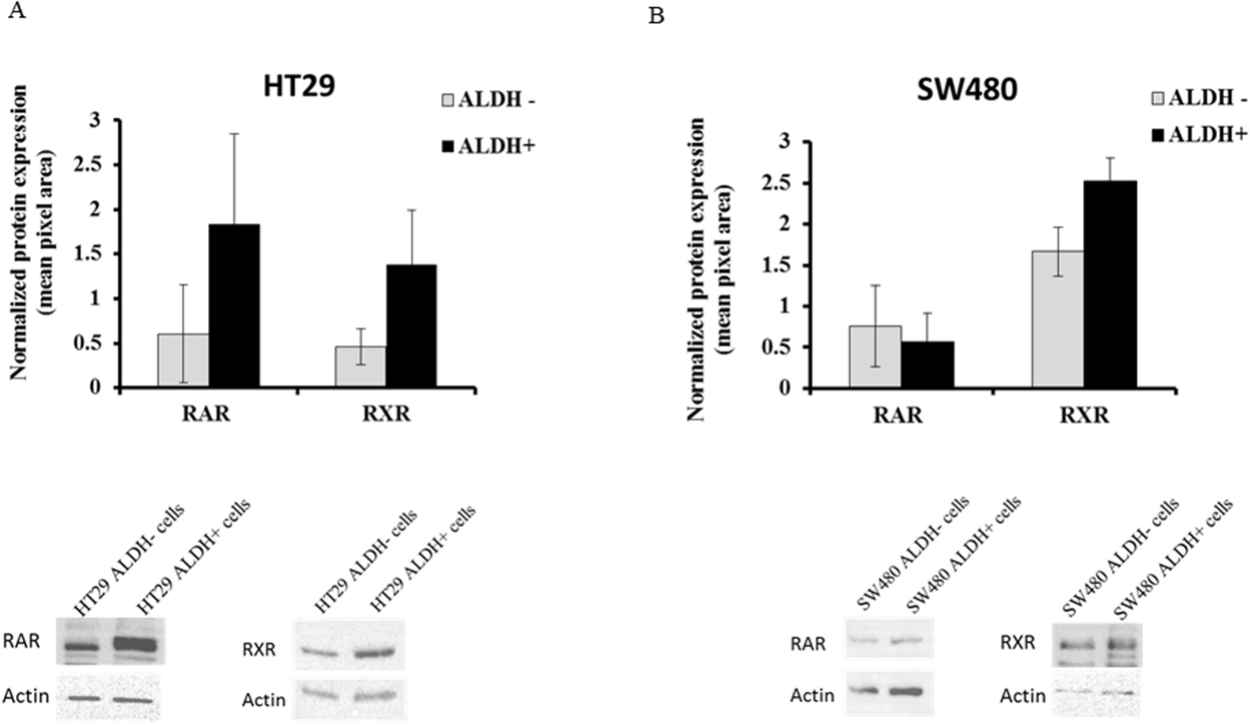
RAR and RXR receptor protein expression in ALDH+ and ALDH- cell populations ALDH+ and ALDH- cells were analyzed by the ALDEFLUOR assay and sorted as separate populations using the BD FACSAria II Flow Cytometer. Sorted cells were pelleted and protein was extracted to run on an SDS-PAGE gel and in western blot analysis. Both RAR and RXR protein expression was analyzed on the sorted **(A)** HT29 cells and **(B)** SW480 cells. The data represents the average densitometry values of each sample probed for RAR or RXR and normalized to the corresponding actin. The western blot images are representative blots from one experiment done on sorted ALDH- and ALDH+ cells from HT29 and SW480 cells and probed for RAR and RXR. Three independent sets of sorts were performed and analyzed; error bars represent ± SEM.

To further analyze protein expression of RXR and RAR in the CRC cell lines, ALDH+ cells and ALDH- cells were sorted from the HT29 and SW480 cells using the ALDEFLUOR assay. Western blot analysis of HT29 cells showed increased expression of both retinoid receptors in ALDH+ cells as compared to ALDH- cells (Figure 3). This is not as clear in the case of SW480 cells. Even so, we show by immunofluorescence analysis of primary colonic tissues that there is co-staining of both RAR and RXR with ALDH1A1 (see Figure 1).

### Effect of ATRA on cell proliferation

To investigate effects of RA ligands on cell growth, we treated colon cancer cell lines (HT29 and SW480) with ATRA. The IC50 values were 10 μM ATRA for HT29 cells and 100 μM ATRA for SW480 cells, as we previously reported [14, 15].

**Figure 4:**
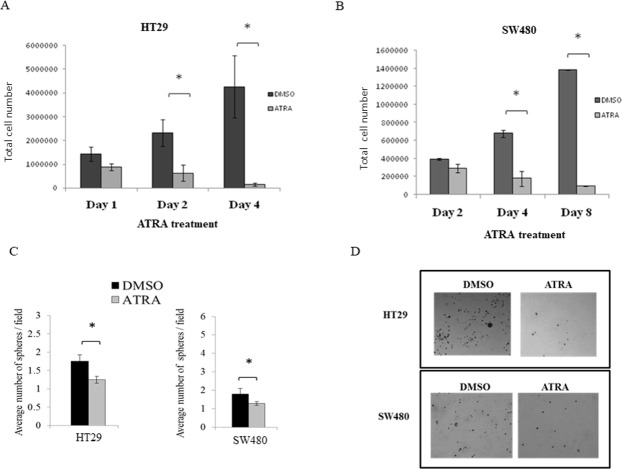
ATRA treatment of both HT29 and SW480 colon cancer cell lines inhibited cell proliferation and decreased sphere formation **(A)** HT29 and **(B)** SW480 cells were treated with the IC50 values of ATRA over a time course and ATRA treatment decreased cell proliferation over time. Cells were trypsinized from each well at each time point and counted using trypan blue exclusion. Cell number at each time point was plotted on the graphs for these experiments. This experiment was done in quadruplicate and error bars represent ± SEM, ^*^ p < 0.05. **(C)** Cells were serum starved for 24 hours and then treated with the IC50 value of ATRA for the designated time points for each cell line (see Materials and Methods). Then single cells were plated for soft agar assay to measure sphere formation after 10 days. ATRA treatment significantly decreased the number of spheres formed in HT29 and SW480 cells. **(D)** Representative images of sphere formation are shown from the last time point of ATRA treatment for each cell line. The numbers of spheres were counted from four wells of each replicate experiment. Experiments were performed in triplicate and error bars represent ±SEM, ^*^ p < 0.05. Images were taken on a phase-contrast microscope using the brightfield setting under a 10x objective.

In time course experiments, we found that cell proliferation in both lines significantly decreased when exposed to ATRA (Figure 4A, 4B). Thereafter, HT29 and SW480 cells were treated with the IC50 values of ATRA in order to assess effects of RA signaling on the SC population via the ALDEFLUOR assay, neuroendocrine cell differentiation, and on anchorage-independent growth.

### Effect of ATRA on sphere formation

Given these effects of RA signaling on proliferation, SC population size and NEC differentiation, we next investigated whether ATRA treatment affects sphere formation. We surmised that if the cells are induced to differentiate by ATRA treatment, then sphere formation should be reduced. Indeed, HT29 cells pre-treated with ATRA resulted in significantly fewer spheres after four days of pre-treatment (Figure 4C). The size of the spheres formed by HT29 cells, as measured by sphere diameter, was not significantly altered at any time point of pre-treatment (Supplementary Figure 2). ATRA pre-treatment of SW480 cells also resulted in significantly fewer spheres as compared to controls (Figure 4C). Similar to HT29 cells, SW480 cells formed spheres of similar size after treatment, as compared to the controls (Supplementary Figure 2). A representative image of the spheres formed for both HT29 and SW480 cells are shown in Figure 4D.

### Effect of ATRA on the ALDH positive SC population size

**Figure 5:**
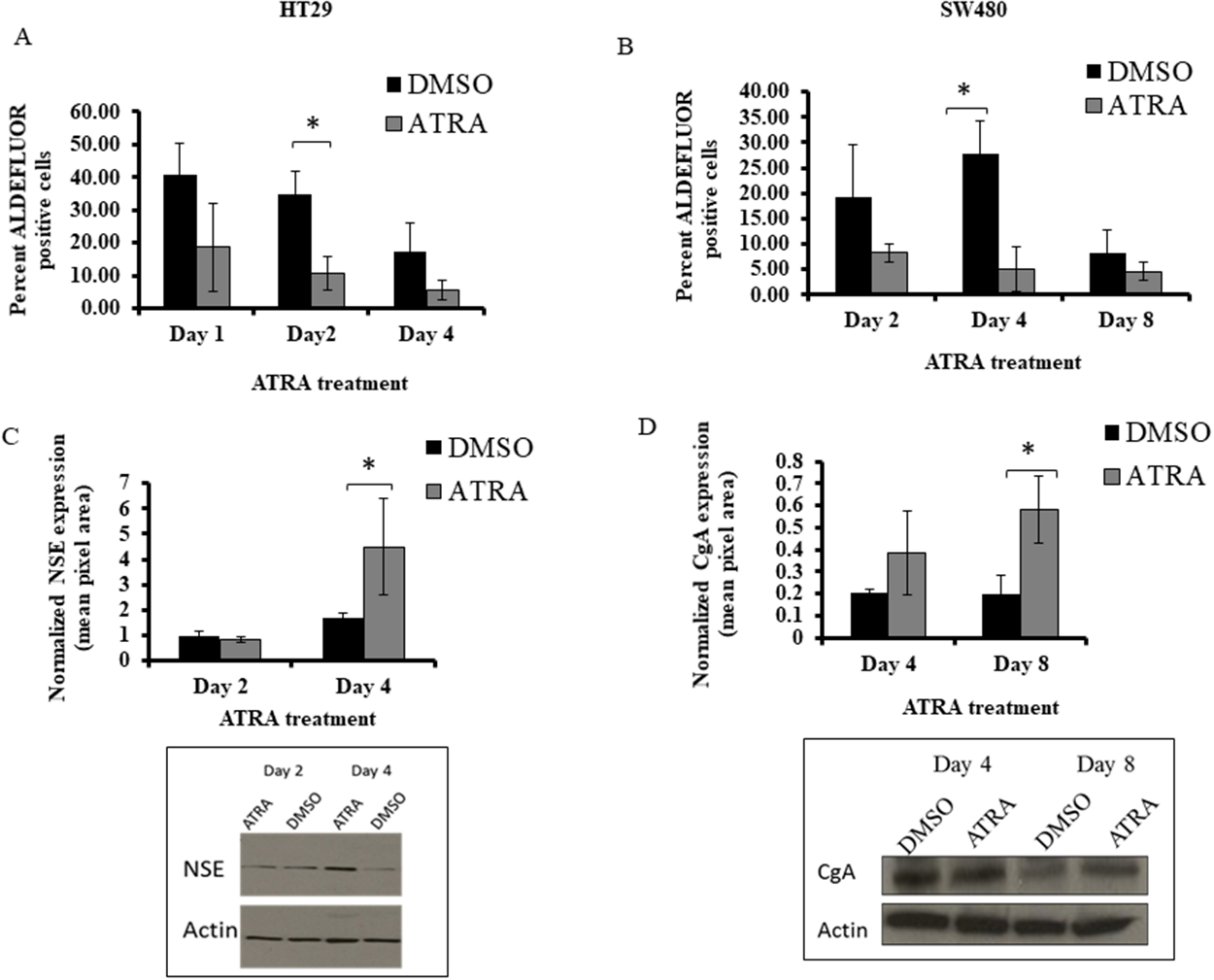
ATRA treatment of HT29 and SW480 cells decreased the percentage of ALDH+ cells and increased NSE and CgA protein expression **(A)** The HT29 cells were treated with 10 μM of ATRA and showed a significant decrease of percent ALDH+ cells at day 2. **(B)** The SW480 cells were treated with 100 μM of ATRA and showed a significant decrease of percent ALDH+ cells at day 4. Note: the decrease in ALDEFLUOR+ cells in DMSO controls is attributed to the decrease in proportion of ALDH+ cells that occurs due to increased cell density of CRC cells that occurs over time in culture [13]. **(C)** In our studies on differentiation, HT29 cells demonstrated a significant increase in NSE expression after 4 days of ATRA treatment. **(D)** SW480 cells demonstrated a significant increase in CgA at day 8 of ATRA treatment. ATRA treatment of the CRC cell lines induced neuroendocrine cell differentiation based on the protein expression of two broad neuroendocrine markers. All western blot data (C and D) represent the average densitometry values of each sample probed for NSE or CgA and normalized to the corresponding actin. The western blot images are representative blots from one experiment done on HT29 and SW480 ATRA treated cells and probed for NSE and CgA. All experiments were done in triplicate and error bars represent ±SEM, ^*^ p < 0.05.

Because RA receptors appear to be selectively expressed in ALDH+ SCs, we investigated whether the addition of exogenous ATRA would specifically change the ALDH+ SC population size. Both cell lines showed a significant decrease in ALDEFLUOR+ cells. This decrease occurred after two days of ATRA treatment of HT29 cells (Figure 5A), and after four days of ATRA treatment of SW480 cells (Figure 5B).

### Effect of ATRA on differentiation of SCs into NECs

Because one way to characterize SCs is by their ability to differentiate into various cell lineages, we analyzed the effects of ATRA on differentiation into NEC cells. We did this by western blot analysis for expression of NSE and CgA proteins, both of which are universal markers for NECs. Our previous publication showed that there are differential levels of expression of NE markers between HT29 and SW480 cells, and that is why different markers can be used to capture the majority of NE cells in a particular cell line [12]. We also observe, by immunofluorescence staining, co-expression of retinoid receptors with CgA in normal colonic crypt cells (Supplementary Figure 3) We found that ATRA treatment of HT29 cells led to a significant increase in NSE expression after 4 days of ATRA treatment (Figure 5C). SW480 cells exhibited a significant increase in CgA expression upon ATRA treatment (Figure 5D). These increases in ATRA-induced SC differentiation into NECs occurred in parallel with the decrease in cell proliferation and the decrease in size of the ALDH+ SC population.

### Effect of CYP26A1 inhibitor liarozole dihydrochloride on HT29 and SW480 cells

**Figure 6:**
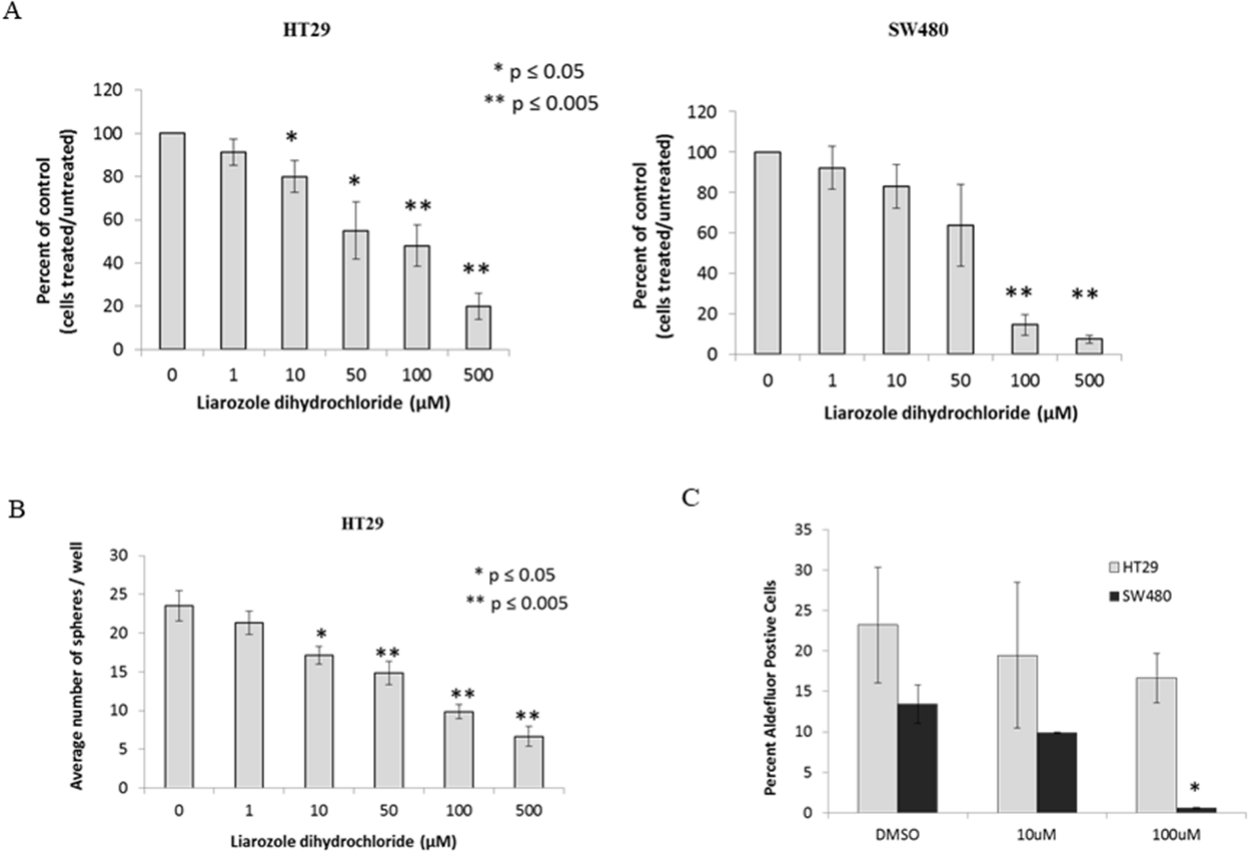
CYP26A1 inhibitor liarozole treatment of HT29 and SW480 colon cancer cell lines decreases cell proliferation, sphere formation and ALDH+ CSCs **(A)** HT29 and SW480 cells treated with liarozole dihydrochloride inhibited cell proliferation in a dose-dependent manner. Cells were trypsinized from each well at each time point and counted using trypan blue exclusion. The average percent of control was plotted on the graphs for these experiments. This experiment was done in triplicate and error bars represent ± SEM, ^*^ p < 0.05. **(B)** HT29 cells were treated with liarozole dihydrochloride (0-500 μM) for three days and plated as single cells to measure colonosphere formation. With an increase in liarozole dihydrochloride concentrations, the number of colonospheres formed decreased. This experiment was done in triplicate and error bars represent ± SEM, ^*^ p < 0.05. Because SW480 cells demonstrate poor sphere formation, they were not assayed. **(C)** HT29 and SW480 cells were treated with a low and high dose of liarozole dihydrochloride and ALDEFLUOR assay was performed to measure changes in ALDH+ population of cells. With the high dose of liarozole dihydrochloride, there is a significant decrease in the percent ALDH+ cells, in the SW480 cells and a decreasing trend in the HT29 cells. This experiment was done in triplicate and error bars represent ± SEM, ^*^ p < 0.05.

**Figure 7:**
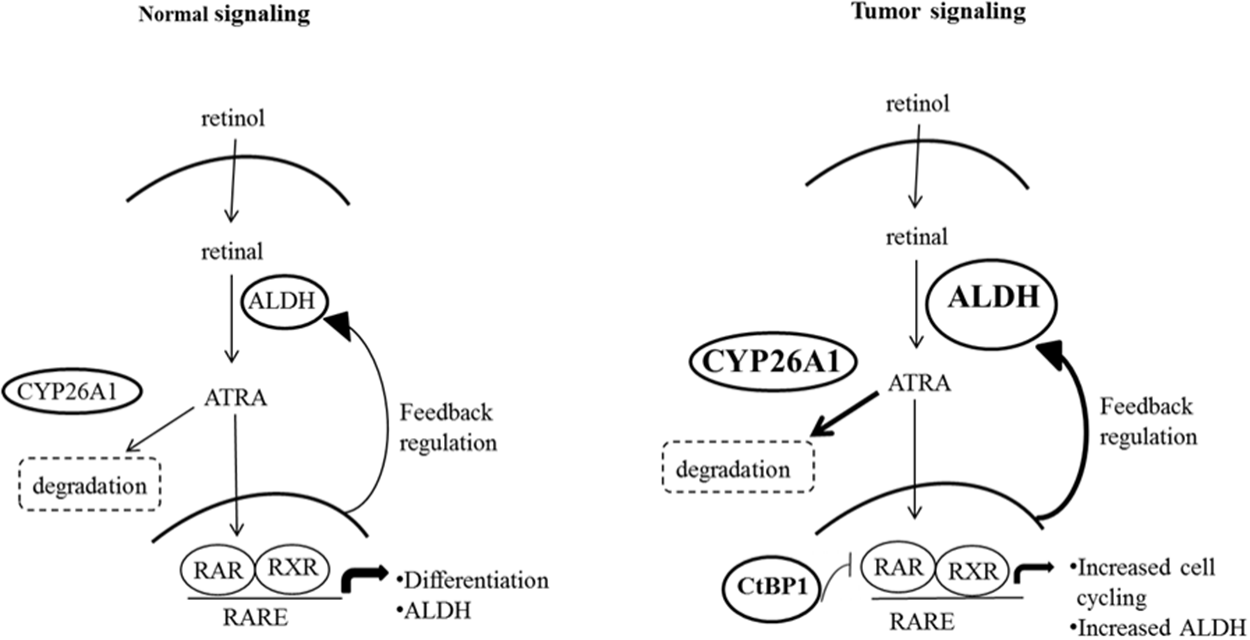
A comparison of normal and malignant colon tissues reveals differences in the expression of several key components in the retinoic acid (RA) signaling pathway In the conversion of retinol to retinal a feedback regulatory mechanism normally maintains homeostatic levels of ALDH (the enzyme that metabolizes retinal into all-trans retinoic acid - ATRA). Through this mechanism, ALDH levels are tightly controlled by intracellular levels of retinoic acid agonists, particularly ATRA [18]. Intracellular ATRA levels are maintained by the enzyme CYP26A1 which catalyzes ATRA degradation. Specifically, RA signaling regulates ALDH via the binding of ATRA to retinoic acid receptors RXR and RAR that transcriptionally control ALDH gene expression. In tumor cells, several components of this pathway are increased, including ALDH, CYP26A1 and CtBP1. Increased ALDH in tumors can be explained by the feedback mechanism whereby increased CYP26A1 enzyme activity leads to ATRA degradation, and decreased ATRA levels then lead to increased ALDH via feedback controls. Specifically, decreased ATRA leads to less RA signaling through RXR- and RAR-based transcriptional control of ALDH. Moreover, we found that tumor cells have increased CtBP1, which acts as a transcriptional repressor of the RAR/RXR/RARE transcriptional complex. Transcriptional repression by CtBP1 not only leads to increased ALDH expression, but also up-regulates expression of genes that promote cell cycling.

Because of the effects of ATRA on ALDEFLUOR+ cells, NEC differentiation, cell proliferation and sphere formation, we analyzed another component of the RA signaling pathway – CYP26A1 [16, 17]. As shown in Figure 7, CYP26A1 is an enzyme that degrades intracellular levels of ATRA and in cancerous cells, CYP26A1 is significantly elevated which fools the cell in thinking there are extremely low levels of ATRA. In turn, the cancerous cell produces more ALDH by increased metabolism of retinal into ATRA. Our final test was to inhibit CYP26A1, by adding exogenous liarozole dihydrochloride, and measure changes in ALDEFLUOR+ cells, cell proliferation, and sphere formation. After treatment with CYP26A1 inhibitor, we saw a decrease in cell proliferation for both cell lines, in a dose dependent manner (Figure 6A). In addition, there was a decrease in sphere formation in a dose dependent manner under anchorage independent conditions (Figure 6B). Finally, when HT29 and SW480 cells were treated with a low and high concentration of CYP26A1 inhibitor, there was a decrease in the ALDEFLUOR+ cells (Figure 6C). Overall, the similar trends and results were seen with the addition of exogenous ATRA and with the inhibition of CYP26A1.

## DISCUSSION

A key finding is that RA signaling mainly occurs through ALDH+ SCs. This conclusion stems from our observation that RXR, RAR are selectively expressed in ALDH+ SCs. For example, our analysis of colonic tissues showed that cells staining positively for RAR and RXR were primarily located in the SC niche at the crypt bottom, where these cells co-stained with ALDH1. Conversely, retinoid receptors did not co-stain with MCM2, a marker for proliferating non-SCs. Our analysis of CRC cell lines also shows that ALDEFLUOR+ cells, which are SCs, tend to have higher levels of RAR and RXR. That RA signaling mainly occurs in ALDH+ SCs, also suggests that RA signaling regulates colonic SC dynamics.

To understand how regulation of ALDH+ SC dynamics might occur, we considered known mechanisms by which ALDH levels are controlled in the RA signaling pathway (Figure 7). Specifically, ALDH expression is regulated via a feedback mechanism involving intracellular ATRA [18–20]. In this mechanism, an increased ATRA concentration leads to feedback inhibition of ALDH1 expression, while decreased ALDH activity decreases conversion of retinaldehyde to RA, which reduces the intracellular ATRA level and lowers activation of RA receptors. Conversely, a decreased ATRA concentration produces the opposite response and generates positive feedback to increase ALDH expression [18]. This feedback mechanism also involves other components downstream of ALDH in the RA pathway, particularly CYP26A1 and CtBP1. CYP26A1 is a cytochrome P450-associated enzyme that metabolizes RA, breaking it down into 4-OH-RA or 4-OXO-RA [16, 17]. CtBP1 is a transcriptional corepressor which acts as a repressor of the RAR/RXR/RARE transcriptional complex. Knowing the mechanisms by which ALDH levels are controlled in the RA signaling pathway, also helps us to understand how dysregulation of the dynamics of ALDH+ SCs might occur.

Indeed, another key finding is that during CRC development, the mechanisms that control ALDH levels become dysregulated. For example, our immunostaining and western blot analyses of matched NCE and CRC tissue samples revealed that RXR and RAR become overexpressed in parallel with increased ALDH. Our analysis of CRC lines showed results similar to those seen for patient tumor samples. Specifically, western blot and immunocytochemical analysis of SW480 and HT29 cells showed increased expression of RA receptors, RAR and RXR, as well as increased expression of CYP26A1 and CtBP1. That RA signaling components are overexpressed in CRC cells and tissues suggests that dysregulation of the mechanisms that control ALDH+ levels contributes to overpopulation of ALDH+ CSCs in CRC.

While our results indicate that the mechanisms that control the dynamics of ALDH+ SCs appear dysregulated in CRC cells, we wanted to see if RA signaling is still functional in CSCs and whether induction of RA signaling can inhibit the growth of ALDH+ CSCs. Because CSCs appear to contain the necessary components for RA signaling, we determined whether RA signaling regulates CSC phenotypes globally – at the level of cell proliferation, CSC population size, sphere formation, and differentiation. We did this by treating HT29 and SW480 cells with ATRA. In both time course and dose response experiments, ATRA treatment decreased cell proliferation of both cell lines. We also observed that ATRA substantially decreased the proportion of ALDEFLUOR+ cells in both cell lines. This finding indicates that the decrease in cell proliferation induced by RA signaling is due to inhibition of growth of the ALDH+ SC population.

That ATRA induced a decrease in cell proliferation, suggested that it was inducing differentiation. Hence, we determined whether activation of RA receptor signaling by ATRA induced SC differentiation toward a NEC phenotype. We chose to specifically analyze NEC differentiation since most crypt NECs reside adjacent to SCs in the SC niche [12, 21]. Indeed, we observed that differentiation toward a NEC lineage was induced in both HT29 and SW480 cell lines upon treatment with ATRA. The promoting effect of ATRA on NEC differentiation in CRC cells is consistent with the fact that ATRA also decreased ALDH+ SC population size.

Since ATRA was found to decrease SC population size and induce differentiation, we surmised that ATRA would also reduce SC colony growth or self-renewal. Hence we treated SW480 and HT29 cells with ATRA and assessed their sphere formation in soft agar assays. Indeed, ATRA-treated HT29 and SW480 cell lines caused a decrease in the number of spheres formed, although no significant changes occurred in the sizes of the spheres. Our findings that ATRA treatment decreases sphere numbers can be explained by our results showing that ATRA reduces ALDH+ SC population size and ATRA increases SC differentiation, which indicates that after being induced to differentiate by ATRA, fewer SCs exist that can generate spheres.

Overall, we have shown that ATRA treatment affects several CSC characteristics and functions. Specifically, treatment of CRC cells with ATRA (i) inhibited proliferation, (ii) decreased ALDH+ SC population size, (iii) induced differentiation of SCs along the NEC lineage, and (iv) reduced sphere formation. These data indicate that RA signaling is functional in colonic CSCs and induction of RA signaling can inhibit the growth of ALDH+ CSC by promoting their differentiation along the NEC lineage.

Our finding that SC signaling components are overexpressed and ALDH+ cells are overpopulated in CRC raises the question: How do alterations in RA signaling pathway components occur in CRC? One answer is that it is due to mutation of the adenomatous polyposis coli (*APC*) gene, which frequently occurs in CRC and is known to drive CRC growth and development. Wildtype APC protein is known to bind to CtBP1 and beta-catenin, and induce degradation of both proteins. In situations where APC is mutated, cells have impaired ability to degrade beta-catenin, which increases TCF4 signaling and proliferation. In the case of CRC, where APC is often mutated, CtBP1 expression levels would also be predicted to be elevated, as our results demonstrate. Overexpression of CtBP1 has also been shown to occur in adenomas from familial adenomatous polyposis patients who are hereditary colon cancer patients carrying germline *APC* mutations [22–24]. Thus, *APC* mutation likely contributes to upregulation of at least two key components of the RA signaling pathway.

Another question arises: How might dysregulation of RA signaling in colonic SCs contribute to overpopulation of colonic SCs that drives CRC development and growth. One explanation for how ALDH becomes overexpressed is based on changes in CYP26A1 and CtBP1 levels when viewed in the context of the RA pathway (Figure 7). In CRC cells, increased levels of CYP26A1 should decrease intracellular ATRA which, via feedback regulation, would increase ALDH expression. In fact, when a CYP26A1 inhibitor was added to HT29 and SW480 CRC cell lines, there was a decrease in ALDEFLUOR+ cells, a decrease in cell proliferation and decrease in sphere formation (Figure 6). Additionally, increased levels of CtBP1 will inhibit normal cell differentiation and upregulate cell cycling genes, which should also increase ALDH expression. Thus, a change in the expression of key components of the RA pathway in CRC cells likely contributes to the overpopulation of ALDH+ SCs and diminishes cell differentiation.

Other factors might also affect the ALDH+ SC population size. For example, expression of different ALDH isoforms may be a factor. Indeed, it has been reported that specific ALDH isoforms, such as ALDH1A1, ALDH1A2, ALDH1A3 and ALDH8A1, play a regulatory role in the initiation and progression of CRC [25]. We also showed that the pattern of ALDH isoform expression changes based on CRC cell density [13]. Changes in expression of different ALDH isoforms might influence the ALDH+ SC population size because the various isoforms have different substrate specificities for RA ligands that could induce changes in RA metabolism and affect the feedback control of ALDH levels. Thus, in future studies, as more information becomes available about these factors, it will be important to understand how they relate to control of ALDH+ SCs based on the RA signaling pathway (Figure 7).

## MATERIALS AND METHODS

### Immunofluorescence

Human colonic tissue sections were analyzed by imunofluorescence as we previously described [2]. Briefly we used anti-ALDH1 (BD Pharmingen, Franklin Lakes, 1:50), anti-RXR-alpha (Santa Cruz, 1:50), anti-RAR-alpha (Santa Cruz, 1:50) and anti-MCM2 (Abcam, Cambridge 1:100) as primary antibodies. The use of human tissues was approved by Institutional Review Boards of Thomas Jefferson University and the Christiana Care Health Services, Inc (FWA00006557).

### Cell culture

HT29 cells obtained from the American Type Culture Collection (ATCC; Manassas, VA) were grown in monolayer cultures and maintained in McCoys medium (GIBCO/Life Technologies) supplemented with 5% fetal bovine serum (FBS), 100 units/mL penicillin and 100μg/mL streptomycin (P/S). SW480 cells obtained from ATCC were maintained in Leibovitz's 15 (L-15) medium (GIBCO/Life Technologies) supplemented with 5% FBS and Penicillin/Streptomycin. All cell cultures were maintained at 37°C in humidified air at 5% CO_2_. Culture medium for all cell lines was changed every 48 hours. The cell lines were routinely tested for mycoplasma using a Universal mycoplasma detection kit (ATCC), and all experiments were carried out with cells cultured within eight - ten passages before earlier freeze down were brought up and used in additional experiments.

### Immunocytochemistry (ICC)

ICC was done using SW480 and HT29 cells between passages 6-10. ICC was done in 8-well chamber slides from Lab tek II. SW480 and HT29 cells were fixed with 4% paraformaldehyde for 40 minutes and then, based on the antibody used the protocol changed. For ALDH1, the cells were then blocked with 1% BSA, 10% serum, 0.2% triton and PBS. For RXR alpha and RAR alpha, 0.2% triton extraction for 20 minutes was done first and the cells were then blocked with 1% BSA, 10% BSA and PBS. Blocking was done overnight at 4 degrees, followed by overnight primary antibody incubation at 4 degrees. Primary antibody was washed off using blocking buffer three times for 10 minutes each time. Secondary antibody, Alexa flour goat anti-rabbit, was used at a dilution of 1:1000 and incubated at room temperature for 1.5 hours. After incubation with secondary antibodies, three washes were done with blocking buffer for 10 minutes. Cells were dried and later stained with SlowFade® Gold antifade DAPI and sealed with coverslips.

### Protein extraction and western blots

Cells were trypsinized, pelleted and lysed with RIPA buffer (0.1% triton, 0.5% sodium deoxycholate, 0.1% SDS, 50 mM Tris HCl and 150 mM NaCl). Protease inhibitor was added to the RIPA buffer just before protein extraction. Cells were then vortexed to break up the pellet and incubated on ice for 30 minutes. Western blot analysis was done for HT29 cells, SW480 cells, and three matched (patient normal and patient tumor) pairs of tissues samples. Normal human colon samples and carcinoma samples were obtained from Christiana Care Hospital after patient consent under an approved IRB protocol. SDS-PAGE was done using polyacrylamide gels (10%; Lonza) and a Bio-rad gel electrophoresis apparatus (2 hours at 120 volts). Protein concentrations were determined using BCA protein assay kits (Pierce, cat # 23227). Protein was transferred to a PVDF membrane (0.45μm, Thermo Scientific, cat # 88518). For analysis of ALDH1 (BD Transduction Labs, cat # 611195), RXR-alpha (Abcam, Cambridge, MA), CtBP1(Abcam), Neuron specific enolase (NSE) (Novus Biologicals, cat# NB-110-58870) and CgA (Abcam), membranes were blocked with 3% BSA in Tris-Buffered Saline with 0.1% Tween 20 (TBST) overnight at 4°C on a shaker. For CYP26A1 (Abcam) and RAR-alpha (Santa Cruz Biotechnology Inc, Dallas, TX) membranes were blocked with 5% milk overnight at 4°C on a shaker. Both primary and secondary antibodies (1:20,000) were prepared in 3% BSA in TBST for ALDH1 (1:300), RXR-alpha (1:500), CtBP1 (1:500), NSE (1:10,000) and CgA (1:1000), and in 5% milk in TBST for CYP26A1 (1:500) and RAR-alpha (1:800). Primary antibody incubation was done at 4°C overnight and then membranes were washed three times with 0.1% TBST for 10 minutes. Secondary antibody incubation was done for 1 hour at room temperature on a shaker and then washed three times with 0.1% TBST for 10 minutes. SuperSignal West Dura Chemiluminescent Substrate (Thermo Fisher Scientific; cat# 34075) was used to detect horseradish peroxidase (HRP) activity and blots were imaged using the Syngene imaging system (Syngene, Frederick, MD). Densitometry was done using the GeneTools software (Syngene).

### ALDEFLUOR assay

Theprotocol followed the manufacturer instructions (STEMCELL Technologies, Vancouver, BC, CANADA) with previously described modifications [12, 13]. Briefly, cells were grown to 80% confluence and detached using 0.25% Trypsin-EDTA (Fisher Scientific). Cells were incubated for 40 minutes at 37°C with ALDEFLUOR reagent. Cell were resuspended in 500 μL ALDEFLUOR buffer and transferred to a BD round bottom tube using a 50 μm cell strainer (BD Biosciences) and analyzed on a BD FACSAria II Flow Cytometer.

### Cell proliferation

All cells were plated at a concentration of 20,000 cells/well of a 24 well plate (four wells per cell line). Medium was replaced every other day. Cells were detached using 0.25% Trypsin-EDTA (Life Technologies) at days 1, 3 and 5, and counted manually with a hemocytometer. Cells were mixed in a 1:1 ratio with Trypan blue (Fisher Scientific) so reported cell counts include the total cell numbers. This was repeated three times and average values were graphed.

### Time course for ATRA

SW480 and HT29 cells were plated at 100,000 cells/well of a 6 well tissue culture plate. Cells were incubated for 24 hours to allow attachment and then serum starved for 24 hours. At the time of treatment, cells were 40-50% confluent. SW480 cells were treated with 100 μM ATRA and HT29 cells were treated with 10 μM ATRA. The IC50 values used for these experiments have been published by our lab previously [14, 15]. These concentrations were determined from the dose response curves for each cell line for each drug. SW480 and HT29 cells were treated for the specified number of days given in results. DMSO (0.1%) was used as a vehicle control for ATRA-treated cells. Cells were counted at each time using a hemacytometer and trypan blue exclusion. Medium with ATRA was changed every 2 days. Each experiment had three replicates of drug treatment and three of vehicle. The final time course graph was plotted based on the average of three individual experimental sets.

### Dose curve for liarozole dihydrochloride

SW480 and HT29 cells were plated at 100,000 cells/well of a 6-well tissue culture plate. Cells were incubated for 24 hours to allow attachment and them serum starved for 24 hours. At the time of treatment, cells were 40-50% confluent. HT29 and SW480 cells were treated with either vehicle control (distilled water – 0 μM), or CYP26A1 inhibitor (Liarozole 1μM, 10μM, 50μM, 100μM, 500μM) for 72 hours [16, 17]. Cells were trypsinized and counted at each concentration, using a hemocytometer and trypan blue exclusion. Experiments were done in quadruplicate and all p-values were determined as a percent of control (treated cells vs untreated cells (vehicle control)).

### Soft agar assay for spherogenic potential

This assay was performed as we previously published [15]. Briefly, low-melting agar (2%) and McCoys medium containing 10% FBS (v/v), 100 U/ml penicillin (P) and 100μg/ml streptomycin (S) in 10 mM citrate buffer were mixed in a 1:1 volume ratio, yielding a final concentration of 1% agar-media solution. This solution was poured into each well of a 24 well plate (Griener, Monroe, NC) and allowed to solidify. A second layer containing 0.25% agar in McCoys medium with 5,000 ATRA treated HT29 cells / well was poured over the first layer of agar and allowed to solidify. When the second layer had solidified, McCoys medium was added to each well. For ATRA treated SW480 cells, the same procedure was followed as with the HT29 cells except for the use of L15 medium. Medium was changed every two days and cultures were allowed to grow for two weeks before being fixed and visualized on either a phase microscope if stained with 0.05% crystal violet or a fluorescent microscope if stained with a green fluorescent nuclear acid stain called Syto13 (Life Technologies, Carlsbad, CA cat # S7575).

### Colonosphere assay

This assay was done according to our previously published protocol [12]. Basically, untreated and liarozole dihydrochloride treated HT29 cells were plated at a cell density of 200 cells/100μl of sphere formation medium and allowed to grow for 14 days. The total number of spheres per well were counted and the average numbers are presented in the graph.

### Statistical analysis

All statistics were performed using Student's t-test using Microsoft excel or one-way ANOVA using Graph Pad Prism software analysis.

## CONCLUSIONS

In the quest to understand how ALDH+ SCs become overpopulated in cancer, this is among the first reports looking at the relationship between ALDH+ colonic SCs and retinoid receptors – RAR and RXR – and the co-localization of the receptors with ALDH. In conclusion, our results indicate that RA signaling regulates the dynamics of colonic CSCs and dysregulation of RA signaling contributes to CRC development. Thus, RA signaling offers a potential therapeutic target by inducing increased differentiation and apoptosis of colonic CSCs. This approach would employ RA agents that restore normal tissue homeostasis rather than trying to induce cytotoxicity as occurs with conventional systemic anti-cancer treatments. Indeed, ATRA has been used as a differentiation agent for *in vitro* studies and for clinical cancer therapy [26–29]. For example, in breast cancer, it has been shown that ATRA induced cell differentiation in breast cancer cells and sensitized the ALDH+ breast cancer cells to chemotherapeutics [30–33]. In acute promyelocytic leukemia, RA agents are highly efficacious and even curative [34]. Our findings – that RA signaling acts through ALDH+ colonic CSCs and that ATRA can decrease SC numbers and sphere formation via differentiation – may provide clues as to how RA agents in combination with other SC-targeting therapies may be developed into new more effective approaches to the treatment of advanced CRC.

## SUPPLEMENTARY MATERIALS FIGURES



## Abbreviations

ALDH: Aldehyde dehydrogenase
ATRA: all-trans retinoic acid
CgA: Chromogranin A
CRC: colorectal cancer
CSC: cancer stem cell
NCE: normal colonic epithelium
NE: neuroendocrine
NSE: neuron specific enolase
RA: retinoic acid
RAR: retinoic acid receptor
RXR: retinoic X receptor
