# Supplementary material for: The anti-cancer effect of retinoic acid signaling in CRC occurs via decreased growth of ALDH+ colon cancer stem cells and increased differentiation of stem cells
Source: Oncotarget. 2018 Oct 5;9(78):34658–69. doi: 10.18632/oncotarget.26157 (PMC6205182; doi:10.18632/oncotarget.26157)
Supplement: Supplementary file 1 [file oncotarget-09-34658-s001.pdf]

The anti-cancer effect of retinoic acid signaling in CRC occurs via decreased growth of ALDH+ colon cancer stem cells and increased differentiation of stem cells

SUPPLEMENTARY MATERIALS

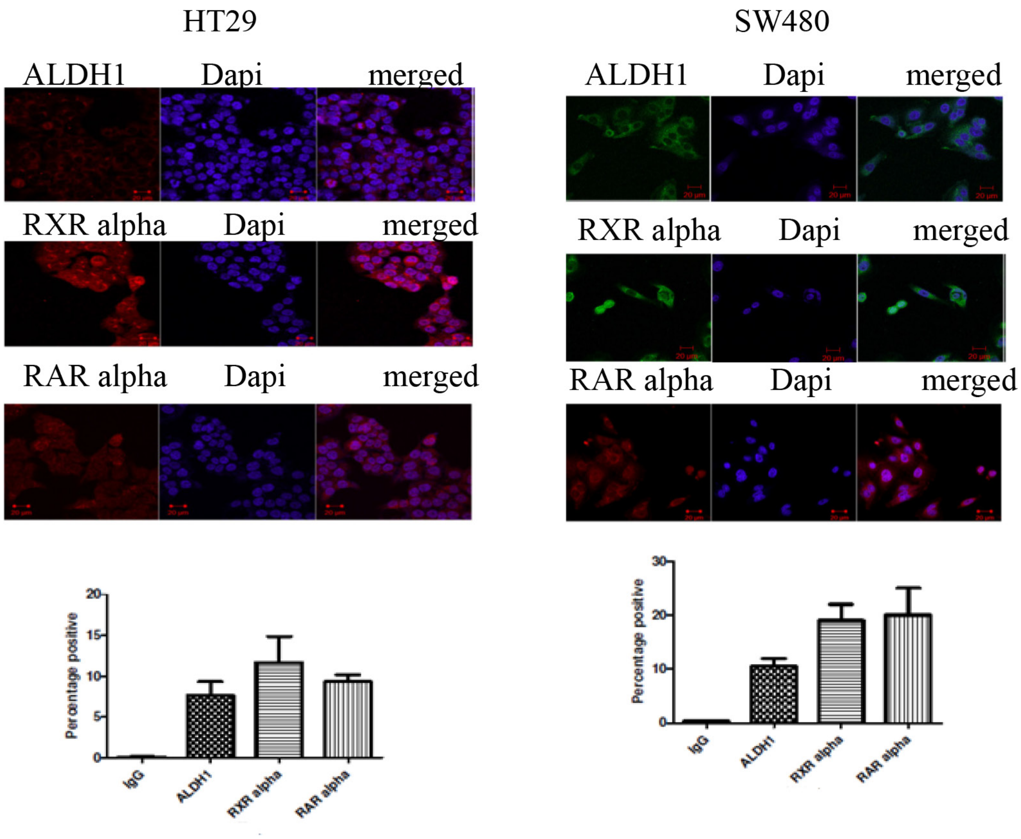

Supplementary Figure 1: CRC cell lines express ALDH1 and retinoid receptors. The expression of retinoid receptors and ALDH1 in HT29 cells and SW480 cells. Immunocytochemistry was done on fixed cells to show nuclear localization of the retinoid receptors and cytoplasmic localization of ALDH1. Quantification of all staining was done by random field counts, and average positive staining was graphed with ± SEM.

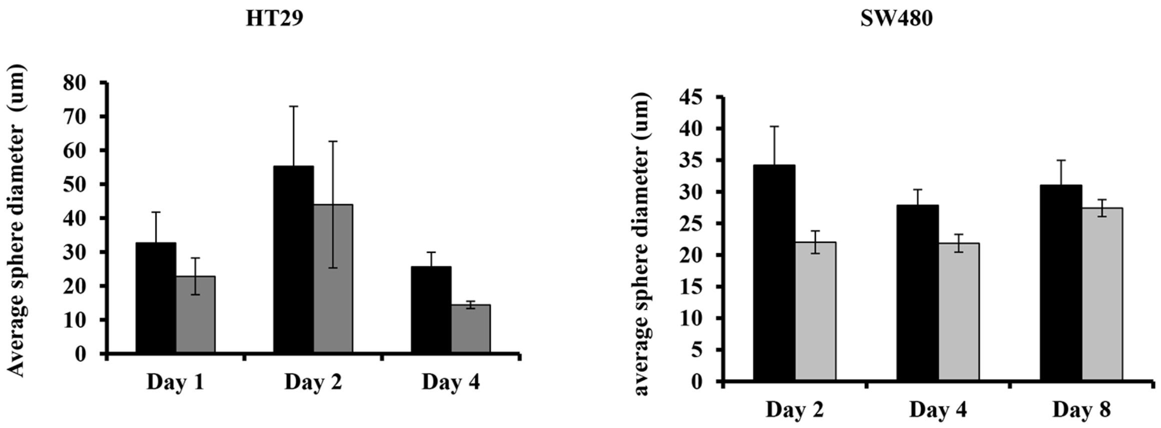

Supplementary Figure 2: ATRA treatment of HT29 cells and SW480 cells did not change the sizes of the colonospheres. Cells were serum starved for 24 hours and then treated with the IC50 value of ATRA for the designated time points for each cell line (see Materials and Methods). Then single cells were plated for soft agar assay to measure sphere formation after 10 days. ATRA treatment did not change the size of the spheres formed in HT29 and SW480 cells. All sphere sizes were measured as diameters in microns. Experiments were performed in triplicate and error bars represent ±SEM, * $p < 0.05$.

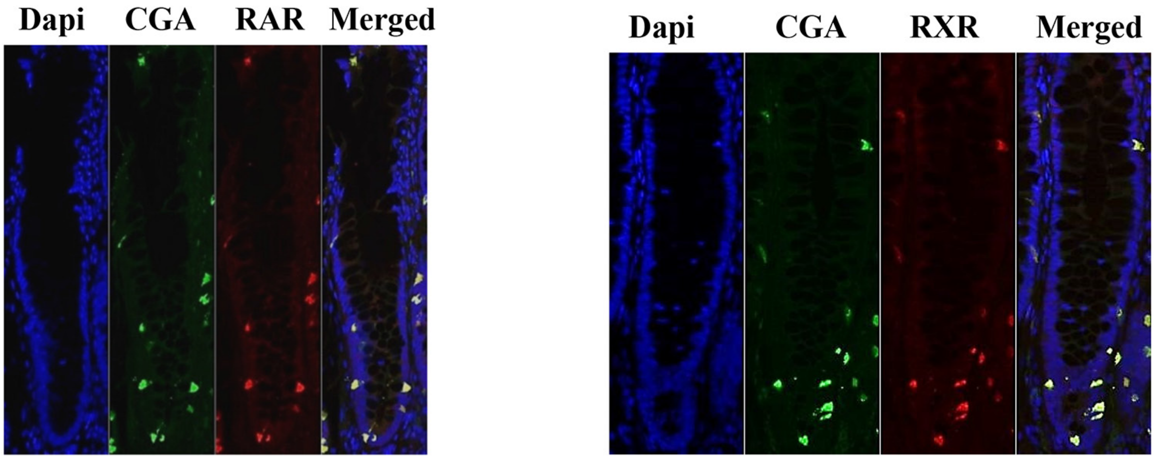

Supplementary Figure 3: Co-staining of retinoid receptors with chromogranin (CgA). Immunofluorescence staining results shows co-expression of RAR and RXR with CgA in normal human colonic crypt cells.
